# Plant Extracts Activated by Cold Atmospheric Pressure Plasmas as Suitable Tools for Synthesis of Gold Nanostructures with Catalytic Uses

**DOI:** 10.3390/nano10061088

**Published:** 2020-06-01

**Authors:** Anna Dzimitrowicz, Piotr Cyganowski, Pawel Pohl, Weronika Milkowska, Dorota Jermakowicz-Bartkowiak, Piotr Jamroz

**Affiliations:** 1 Department of Analytical Chemistry and Chemical Metallurgy, Wroclaw University of Science and Technology, Wybrzeze St. Wyspianskiego 27, 50-370 Wroclaw, Poland; pawel.pohl@pwr.edu.pl (P.P.); wmilkowska@o2.pl (W.M.); piotr.jamroz@pwr.edu.pl (P.J.); 2 Department of Process Engineering and Technology of Polymer and Carbon Materials, Wroclaw University of Science and Technology, Wybrzeze St. Wyspianskiego 27, 50-370 Wroclaw, Poland; piotr.cyganowski@pwr.edu.pl (P.C.); dorota.jermakowicz-bartkowiak@pwr.edu.pl (D.J.-B.)

**Keywords:** gold nanoparticles, non-thermal plasmas, botanical extracts, 4-nitrophenol, catalysis

## Abstract

Because cold atmospheric pressure plasma (CAPP)-based technologies are very useful tools in nanomaterials synthesis, in this work we have connected two unique in their classes approaches—a CAPP-based protocol and a green synthesis method in order to obtain stable-in-time gold nanoparticles (AuNPs). To do so, we have used an aqueous *Gingko biloba* leave extract and an aqueous *Panax ginseng* root extract (untreated or treated by CAPP) to produce AuNPs, suitable for catalytical uses. Firstly, we have adjusted the optical properties of resulted AuNPs, applying UV/Vis absorption spectrophotometry (UV/Vis). To reveal the morphology of Au nanostructures, transmission electron microscopy (TEM) in addition to energy dispersive X-ray scattering (EDX) and selected area X-ray diffraction (SAED) was utilized. Moreover, optical emission spectrometry (OES) in addition to a colorimetric method was used to identify and determine the concentration of selected RONS occurring at the liquid-CAPP interface. Additionally, attenuated total reflectance Fourier transform-infrared spectroscopy (ATR FT-IR) was applied to reveal the active compounds, which might be responsible for the AuNPs surface functionalization and stabilization. Within the performed research it was found that the smallest in size AuNPs were synthesized using the aqueous *P. ginseng* root extract, which was activated by direct current atmospheric pressure glow discharge (dc-APGD), generated in contact with a flowing liquid cathode (FLC). On the contrary, taking into account the aqueous *G. biloba* leave extract, the smallest in size AuNPs were synthesized when the untreated by CAPP aqueous *G. biloba* leave extract was involved in the Au nanostructures synthesis. For catalytical studies we have chosen AuNPs produced using the aqueous *P. ginseng* root extract activated by FLC-dc-APGD as well as AuNPs synthesized using the aqueous *G. biloba* leave extract also activated by FLC-dc-APGD. Those NPs were successfully used as homogenous catalysts for the reduction of 4-nitrophenol (4-NP) to 4-aminophenol (4-AP).

## 1. Introduction

Currently, a significant increase in the interest of nanomaterials, especially noble-metal nanostructures among others is noted. These metallic nanoparticles (NPs) are widely applied in different technologies due to a higher number of atoms on their surface compared to their macroscopic forms. Because of a high stability as well as an ability to absorb visible light caused by the plasmonic resonance [1], gold nanoparticles (AuNPs) are used in cancer treatments (e.g., in the plasmonic photothermal therapy (PTTT) as photoabsorbing agents) [2], in the optics (e.g., as sensors) [3], in the analytical chemistry (e.g., as detectors) [4], as well as in the inactivation of the pathogenic microorganisms (e.g., as antimicrobial agents) [5]. An extraordinary high surface to volume ratio of AuNPs makes them also a material, which catalytic activity is very tempting in a number of reactions [6,7,8].

A few scientific reports provide examples of AuNPs applications as nanocatalysts for different and often challenging reactions. Recently, it has been proven that utilization of AuNPs increases the efficiency of chemical transformations, which includes degradation of for example, toxic organic pollutants (such as 4-nitroaniline and 4-nitrophenol (4-NP)) [9,10,11], dyes (such as Congo red, acridine orange, bromothymol blue, phenol red, methylene blue and rhodamine B) [10,12,13], biomolecules (such as estradiol (E2)) [14] and monosaccharides (such as arabinose) [15]. Although the literature indicates that utilization of AuNPs immobilized on different supports should be preferred from a practical point of view [15,16,17,18,19], the homogenous catalysts containing AuNPs often exceed the catalytic efficiency of heterogeneous ones [9,10,11,12,13,14]. It is usually attributed to their ability to be active on a “molecular level” [20]. However, the use of AuNPs as a homogenous catalyst involves number of issues, arising from a limited stability thereof, as well as their tendency to aggregate. For these reasons, a special attention should be paid to the approach of AuNPs synthesis [21].

There is a wide range of wet-chemistry-based methods suitable for AuNPs synthesis [22]. The most popular protocol was developed in 1951 by Turkevich [22]. In this approach, sodium citrate acts as a stabilizing and capping agent and leads to obtain spherical AuNPs, ranging in size from 10 to 20 nm [22]. Another example of the wet-chemistry-based protocol was proposed in 1994 by Brust. In this method, tetrahydroborate (THB) acts as a reducing agent while alkenothiol acts as a capping agent. As a result, 1.5–5.0 nm AuNPs are produced [22]. The above-mentioned methods for the AuNPs syntheses are simple and widely utilized. However, the chemical reducing and capping agents may be an additional source of contamination, which, due to the potential catalytic decomposition of harmful pollutants may lead to an additional purification of the Au nanostructures after their synthesis. A remedy for these inconvenient drawbacks might be a substitution of wet-chemistry-approaches by green synthesis methods or cold atmospheric pressure plasma (CAPP)-based methods in order to produce AuNPs.

In the green synthesis of NPs, nanostructures precursors are reduced by active compounds present in plants, bacteria or fungi. In 2019 and 2020 several plant species were used in AuNPs synthesis. This included, for example, *Rosmarinus officinalis* [23], *Salvia hispanica* [24], *Eucalyptus globulus* [23], *Mentha piperita* [24], *Melisa officinalis* [24], *Pimpinella anisum* [25], *Jasminum auriculatum* [26], *Hubert ambavilla* [27], *Lactuca indica* [28], *Solanum nigrum* [29], *Brassica oleracea* [30] and *Commiphora wightil* [31] plant species that were a source of reducing and capping agents. Most of the so-obtained Au nanostructures were applied as catalysts [26,28,29,30] in the different catalytical processes, for example, catalytic reduction of 4-NP to 4-aminophenol (4-AP) [26,29,30] and catalytic reduction of methyl orange [28]. Among different plant species used for the AuNPs synthesis, a special attention was paid to two popular herbs, that is, *Ginkgo biloba* and *Panax ginseng,* both commonly occurring in West-Asia.

*G. biloba* (or maidenhair tree eaves) is a rich source of several active compounds including ginkgolic acids, terpenes trilactiones, flavonoids and proanthocyanidins [32]. Up to now, several research groups have employed *G. biloba* in the AuNPs production [33,34,35] using 5% [35], 20% [33] or 25% [34] aqueous *G. biloba* leaves extracts. The size of the so-obtained Au nanostructures was 18.95 ± 5.95 nm [35], 10–75 nm [33] or 1–40 nm [34], respectively. The resultant AuNPs were applied in the detection of Cr (VI) ions in drinking water [35] or as a colorant in the fabrics [33]. On the other hand, *P. ginseng* (or *Chinese ginseng*) contains at least 40 different ginsenosides compounds of excellent antioxidant properties [36]. For that reason, the aqueous *P. ginseng* root extract has been applied to produce AuNPs, which size ranged from 5 to 350 nm [37,38,39,40]. Unfortunately, in these works [37,38,39,40], the applications of so-obtained AuNPs are not described.

The issue involving the purification process of nanostructures after their production has been resolved in our research group by using the CAPP-based synthesis. In this case, the reduction of the Au (III) ions to their metallic Au (0) form in nanometric scale was caused by the reactive oxygen and nitrogen species (RONS) generated during the non-thermal plasma operation [41,42,43,44]. Because RONS exhibit excellent oxidation-reduction properties, the AuNPs precursor solution may consist only of Au (III) ions [41]. However, having in mind a potential use of AuNPs as homogenous catalysts, capping agents should be introduced into the system anyway to provide their long-term stability and aqueous plant extracts seem to be perfect for this purpose. To connect these two approaches, that is, the green synthesis method and CAPP-based method for the NPs fabrication, we previously examined how the proper CAPP treatment influences on the granulometric properties of AuNPs obtained by using *M. officinalis, M. piperita or S. hispanica* aqueous plant extracts [24]. Unfortunately, we observed the increase in the size of the nanostructures treated by CAPP [24].

The main aim of this work was to develop an innovative and low-cost method, which connects the green synthesis along with the CAPP-based approach for the production of AuNPs with catalytic properties. To reach this goal, we compared the optical and granulometric properties of AuNPs produced using untreated aqueous plant extracts with these activated with proper CAPP and revealed how the proper non-thermal plasma treatment affected the Au nanostructures properties. To the best of our knowledge this is the first work in which CAPP was used to activate aqueous *G. biloba* and *P. ginseng* plant extracts for their further application in the synthesis of the Au nanostructures that are dedicated to the catalytic use in the form of a homogenous catalyst. The resulted Au nanostructures were characterized using UV/Vis absorption spectrophotometry (UV/Vis) as well as transmission electron microscopy (TEM) supported by energy dispersive X-ray scattering (EDX) and selected area electron diffraction (SAED). Additionally, the identification of selected RONS present at the liquid-CAPP interface was performed using optical emission spectrometry (OES). Nevertheless, the concentration of nitrite in the untreated as well as CAPP-treated aqueous plant extracts was determined using a colorimetric method. To identify the reactive compounds, which might be responsible for the AuNPs surface functionalization, the attenuated total reflectance Fourier transform-infrared spectroscopy (ATR FT-IR) was used. Finally, the selected stable-in-time suspensions of AuNPs were used as homogenous nanocatalysts in the model reaction of the catalytic decomposition of 4-NP to 4-AP.

## 2. Materials and Methods

### 2.1. Reagents and Solutions

*G. biloba* leaves powder and *P. ginseng* root powder were purchased from a local Polish company. A tetrachloroauric acid tetrahydrate (HAuCl_4_ × 4H_2_O) was bought in Pol-Aura (Olsztyn, Poland). 4-nitrophenol, as well as sodium borohydrite (NaBH_4_) powders (>99%), were acquired from Sigma-Aldrich Co. (Steinheim, Germany). All chemical reagents were of an analytical grade purity. De-ionized water was used in all experiments.

### 2.2. Plant Extract Preparation

To prepare 2% (m/m) aqueous plant extracts, 2 g of the dry powders of *G. biloba* leaves or *P. ginseng* root were mixed with 98 g of de-ionized water and heated to boil. Then, the mixtures were kept boiling for 10 min. Afterwards, they were filtered using 115 qualitative hard filter papers (Chemland, Stargard Szczecinski, Poland). The resulted 2% (m/m) aqueous plant extracts were stored at 4 °C in the dark for further uses.

### 2.3. Plant Extracts Activation by Cold Atmospheric Pressure Plasmas

**Figure 1:**
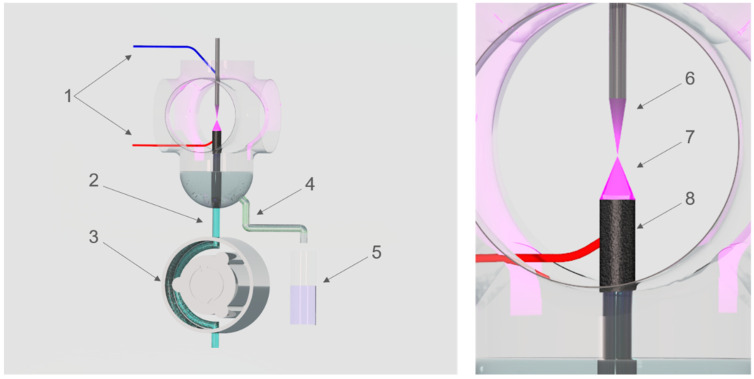
The plant extracts activation by direct current atmospheric pressure glow discharge (dc-APGD) operated in two modes (FLA-dc-APGD or FLC-dc-APGD). (**1**) high-voltage connections; (**2**) untreated plant extract inlet acting as a FLA or a FLC; (**3**) peristaltic pump; (**4**) collector of the activated plant extract; (**5**) container with an AuNPs precursor solution; (**6**) tungsten electrode; (**7**) FLA-dc-APGD or FLC-dc-APGD; (**8**) graphite tube covering a quartz capillary.

To activate aqueous plant extracts, the direct current atmospheric pressure glow discharge (dc-APGD) was applied as a CAPP source. dc-APGD was operated in a high-throughput continuous reaction-discharge system, working in two different modes (Figure 1) [45]. In the first mode, that is, FLA-dc-APGD, dc-APGD was generated in a gap between the surface of a flowing liquid anode (FLA), being an aqueous plant extract solution and a sharpened tungsten cathode. In the second mode, that is, FLC-dc-APGD, dc-APGD was generated in a gap between the surface of a flowing liquid cathode (FLC), being an aqueous plant extract solution and a sharpened tungsten anode. The gap between the FLA (or FLC) and the metallic electrode was ≈ 4.0 mm. In both cases, the aqueous plant extract solutions were delivered to a quartz chamber, applying a four channel peristaltic pump (Masterflex L/S, Cole-Parmer, Vernon Hills, Il, USA), through a graphite/quartz tube (OD = 6 mm). The flow rate of the introduced aqueous plant extracts to the reaction-discharge system was 4.0 mL min^−1^. To provide a HV potential to an electrical circuit, a high voltage generator (Dora Electronics Equipment, Wroclaw, Poland) was used. The discharge current of 50 mA was maintained by applying a 10 kΩ ballast resistor (Tyco Electronics, Berwyn, IL, USA). The voltage contact was provided by a Pt wire attached to the graphite/quartz tube. After applying the FLA-dc-APGD or FLC-dc-APGD treatment of the aqueous plant extracts, they were kept for further analyses or immediately mixed with an AuNPs precursor solution in order to produce Au nanostructures.

### 2.4. Synthesis of Gold Nanostructures Using Untreated as Well as CAPP-Treated Aqueous Plant Extracts

A stock solution (1000 μg mL^−1^) of the Au (III) ions was applied as an Au nanostructures precursor. In order to produce AuNPs using untreated as well as CAPP-activated aqueous plant extracts, a defined volume of the stock solution was immediately mixed with 2% (m/m) aqueous *G. biloba* or *P. ginseng* plant extracts. As a result, the respective reaction mixtures were obtained, in which the final concentration of the plant extract was 1% (m/m) and the concentration of the Au (III) ions was 125, 250 or 500 μg mL^−1^. The produced AuNPs were kept at 4 °C for further characterization and applications.

### 2.5. Characterization of the Optical and Granulometric Properties of Gold Nanostructures

The optical properties of synthesized AuNPs, according to the occurrence of the λ_max_ of the localized surface plasmon resonance (LSPR) absorption band and its absorbance, were determined using UV/Vis absorption spectrophotometry. A Specord 210 Plus (Analytik Jena, Jena AG, Germany) was used to acquire UV/Vis absorption spectra of 25-diluted AuNPs colloidal suspensions. The UV/Vis spectra were recorded in the range from 400 to 700 nm, with a step of 0.2 nm. As a background, de-ionized water was used.

The morphology of produced AuNPs in relevance to their size, shape, crystalline structure and elemental composition was assessed using a Tecnai G^2^ 20 X-TWIN TEM (FEI, Hillsboro, OR, USA) supported by an EDX X-ray microanalyzer and a SAED analyzer (FEI, Hillsboro, OR, USA). To perform TEM, EDX and SAED analyses, a 100 μL of a proper 25-diluted AuNPs colloidal suspension was placed onto a Cu grid (CF400-Cu-UL, GF MICROSYSTEMS, Poznan, Poland) and let to dry.

### 2.6. Studies of the Interactions and Processes Occur at the Extract-Cold Atmospheric Pressure Plasma Interface and Leading to the Plant Extract Activation

The OES was used to identify the RONS in the untreated as well as FLA-dc-APGD or FLC-dc-APGD-treated aqueous *G. biloba* leave or *P. ginseng* root extracts. The OES spectra were acquired in the range of 200 to 900 nm using a Shamrock SR-500i (Andor, UK) spectrometer, equipped with a Newton DU-920P-OE CCD camera. The radiation emitted by CAPP was collimated by an achromatic lens on the entrance slit (10 μm) of the spectrometer. The integration time of the CCD camera was set to 0.10 s.

The concentration of nitrite ions in the analyzed reaction mixtures, containing AuNPs produced using untreated as well as FLA-dc-APGD or FLC-dc-APGD-treated aqueous *G. biloba* or *P. ginseng* extracts, was determined applying a commercial colorimetric kit assay (HANNA Instruments, Olsztyn, Poland), employing for that purpose HANNA HI 96708 spectrophotometer (HANNA Instruments, Olsztyn, Poland). All measurements were carried out according to the protocol suggested by the manufacturer (HANNA Instruments, Olsztyn, Poland).

### 2.7. Gold Nanoparticles Stabilization by Active Compounds Originating from Plant Extracts

The presence of active compounds was detected by ATR FT-IR spectroscopy. A Jasco FT/IR-4700 device (Tokyo, Japan), equipped with a diamond ATR attachment (Tokyo, Japan) was used. ATR FT-IR spectra were recorded in the range of 4000–400 cm^−1^ with 64 scans and resolution of 4 cm^−1^. To carry out ATR FT-IR analyses, one drop of analyzed solutions was placed onto a diamond cell and evaporated under vacuum.

### 2.8. Homogenous Catalysis

Selected samples of AuNPs were used as homogenous nanocatalysts in a model reaction of the reduction of 4-NP to 4-aminophenol (4-AP). The reaction was carried out at 20 °C in a quartz cuvette, where 3 mL of a 0.1 mmol L^−1^ 4-NP solution was introduced. Then, 0.3 mL of a 0.1 mol L^−1^ NaBH_4_ solution was added. Afterwards, 0.3 mL of the analyzed AuNPs colloidal suspension was added to the reaction mixture. The catalytic reaction was monitored using UV/Vis absorption spectrophotometry for each stage of the reaction mixture preparation and each 1 min after the nanocatalyst was introduced until the 4-NP was entirely decomposed. The course of the catalytic reaction was monitored using a Jasco V730 UV/Vis absorption spectrophotometer (Tokyo, Japan); suitable spectra were recorded in the range from 200 to 700 nm.

The catalytic activity of selected AuNPs was estimated by defining a maximum absorbance (λ_max_) of 4-NP over a time. Because a decrease of λ_max_ was proportional to the concentration of 4-NP, the constant rate (*k*) was calculated from the slope of the ln (A_t_/A_0_) graph versus the time t (in s), where A_t_ is the absorbance and A_0_ is the absorbance at the beginning of the process. Then, the k value, expressed in s^−1^, was re-calculated to the mass normalized rate constant *k_m_* (in s^−1^ mg^−1^).

## 3. Results and Discussion 

### 3.1. Optical Properties of Gold Nanostructures

UV/Vis absorption spectra were acquired 60 min after the AuNPs synthesis. Based on the position of the LSPR absorption band in the UV/Vis spectrum, it was possible to assess the optical properties of AuNPs in addition to a preliminary estimation of their size. Typically, for spherical AuNPs, the LSPR absorption band occurs within 520 to 550 nm [45].

**Figure 2:**
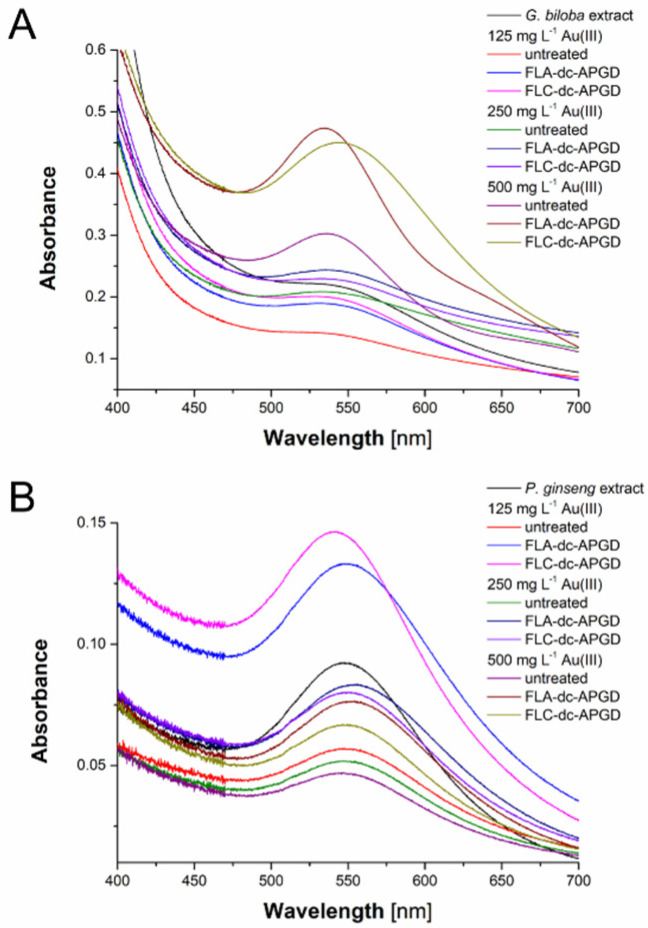
UV/Vis spectra of (**A**) the aqueous *G. biloba* leave extract and (**B**) the aqueous *P. ginseng* root extracts and AuNPs obtained therefrom.

As can be seen from Figure 2 and Table 1, for most of resulted AuNPs the LSPR absorption band shifted towards longer wavelengths with the increasing of concentration of the Au (III) ions in the reaction mixture. It was related to the described in 1908 Mie’s light scatter theory [46]. Based on this, it was concluded that the smallest AuNPs were produced using the reaction mixture consisting of the untreated *G. biloba* aqueous leaves extract and 125 mg L^−1^ of the Au (III) ions. Furthermore, the biggest AuNPs were obtained when the aqueous *G. biloba* leaves extract was activated by FLC-dc-APGD and when the final concentration of the Au (III) ions was 500 mg L^−1^ in the reaction mixture. Taking into account the application of the aqueous *P. ginseng* root extract in the AuNPs synthesis, the smallest AuNPs were produced using the reaction mixture consisting of the aqueous *P. ginseng* root extract treated by FLC-dc-APGD and 125 mg L^−1^ of the Au (III) ions. Additionally, the biggest Au nanostructures were produced when the aqueous *P. ginseng* root extract was activated by FLA-dc-APGD and when the final concentration of the Au (III) ions was 250 mg L^−1^ in the reaction mixture. These observations might be related to the characteristics of the applied discharges. When the aqueous plant extract was activated by FLC-dc-APGD, the surface of the FLC was irradiated by the positive ions from the plasma anode [45,47,48]. On the contrary, while the aqueous plant extract was activated by FLA-dc-APGD, the surface of the FLA was irradiated by the electrons from the plasma cathode [45,47,48]. For that reason, the polarity of electrodes used in those systems might have an impact on the type and concentration of the generated RONS. Moreover, these observations might be associated with the influence of the active compounds present in the applied aqueous plant extracts on the position of the LSPR absorption band in the analyzed UV/Vis absorption spectra [32,36]. In addition, for both types of plant extracts activated either by FLC-dc-APGD or FLA-dc-APGD, the increase in the absorbance values was observed. As was suggested by Badi’ah et al. [49], this increase was likely related to the growth of NPs and connected to the enhanced efficiency of the AuNPs synthesis as compared to the untreated aqueous plant extract.

### 3.2. Granulometric Properties of Gold Nanostructures

To reveal the structural properties of AuNPs obtained using untreated as well as activated by FLA-dc-APGD or FLC-dc-APGD aqueous plant extracts, several experimental techniques including TEM, EDX and SAED were used. 

**Figure 3:**
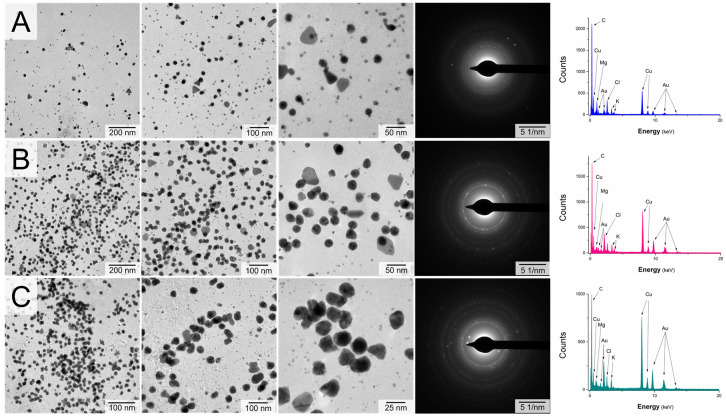
Transmission electron microscopy (TEM) photomicrographs, selected area X-ray diffraction (SAED) patterns and energy dispersive X-ray scattering (EDX) spectra of AuNPs obtained using (**A**) untreated; (**B**) flowing liquid anode (FLA)-dc-APGD-activated; or (**C**) flowing liquid cathode (FLC)-dc-APGD-activated aqueous *G. biloba* leaves extracts. The concentration of Au (III) ions used for the synthesis of biogenic AuNPs was 500 mg L^−1^.

**Figure 4:**
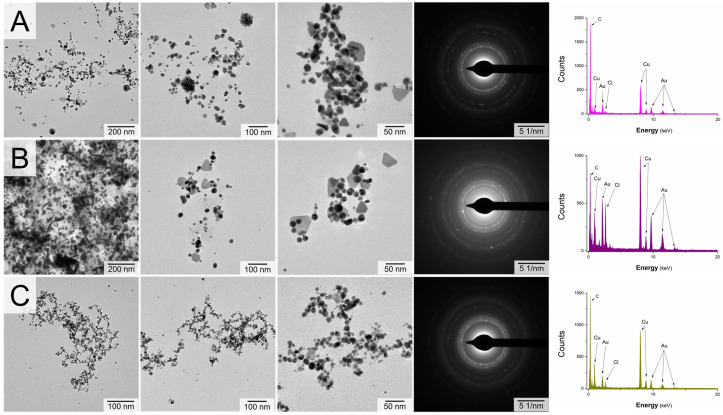
TEM photomicrographs, SAED patterns and EDX spectra of AuNPs obtained using (**A**) untreated; (**B**) FLA-dc-APGD-activated; or **(C)** FLC-dc-APGD-activated aqueous *P. ginseng* root extracts. The concentration of Au (III) ions used for the synthesis of biogenic AuNPs was 500 mg L^−1^.

Figure 3 and Figure 4 display TEM photomicrographs of AuNPs obtained using either untreated aqueous plant extracts (A) or aqueous plant extract activated by FLA-dc-APGD (B) or FLC-dc-APGD (C). Because the aim of the present work was to compare different approaches for the fabrication of biogenic AuNPs, their granulometric properties are also summarized in Table 2. The average size of AuNPs was calculated based on the 300–700 NPs, depending of the well-observed grain boundaries of analyzed nanostructures.

As can be seen from Figure 3 and Figure 4, the application of untreated aqueous plant extracts resulted in the fabrication of mainly spherical AuNPs. A similar effect was observed in the case of the application of plant extracts activated using FLC-dc-APGD. The utilization of FLA-dc-APGD seemed to result in decreasing the content of spherical AuNPs (see Table 2). Accordingly, a greater amount of triangular and rod-like Au nanostructures was observed. These differences were likely linked with the nature of the CAPP source, as explained by the LSPR bands shifts described above (see Section 3.1. for more details). In the case of the aqueous *P. ginseng* extract the smallest in size AuNPs were produced after the plant extract was activated using FLC-dc-APGD (14.2 ± 3.8 nm), while considerably bigger AuNPs (21.3 ± 12.3 nm) were observed in the case of the plant extract activation by FLA-dc-APGD (see Table 2). The effect was opposite in the case of the aqueous *G. biloba* extract because its FLA-dc-APGD activation led to the formation of smaller AuNPs as compared to the FLC-dc-APGD (24.4 ± 4.4 vs. 29.0 ± 3.5 nm). However, considering the standard deviation values (STD) (see Table 2), these sizes could be considered as very close to each other, hence, it was concluded that the average diameter of AuNPs was depended on either the CAPP source or/and the characteristics of the plant extract. This observation was supported by the fact that the plant extract activation by non-thermal plasma slightly increases the average size of resultant AuNPs (Table 2), as compared to the nanostructures fabricated using the untreated aqueous plant extracts. Hence, it was expected that the plant extract activation either by FLC-dc-APGD or by FLA-dc-APGD led to increase the concentration of RONS in the plant extracts, hence, was responsible for their increased reducing potential.

In order to reveal the elemental composition as well as the crystalline structure of the so-obtained Au nanostructures, the EDX and SAED analyses were carried out. In all the acquired EDX spectra (Figure 3 and Figure 4), the presence of Au (from AuNPs), Cu (from the Cu grid), O and C (from organic compounds present in the plant material extracts) and Cl (from the nanostructures precursor) were detected. Additionally, in the case of the AuNPs produced using the aqueous *G. biloba* leaves extract, the presence of K and Mg was also identified. As was suggested by Koczka et al. [50], these elements are present in the leaves of *G. biloba* in high amounts. Based on the EDX patterns, it might be concluded that the green synthesis of biogenic AuNPs was successful in all the described cases. Taking into account the crystalline structure of the produced AuNPs based on the SAED diffractograms, the following d-spacing was noted for all fabricated biogenic AuNPs: 1.26, 1.52, 2.15 and 2.44 Å. This was consistent with the literature data [51,52], proving that the detected nanostructures were indeed AuNPs of the face-centered cubic (fcc) structure.

### 3.3. Nature of Plant Extracts Activated by Proper CAPP Source

**Figure 5:**
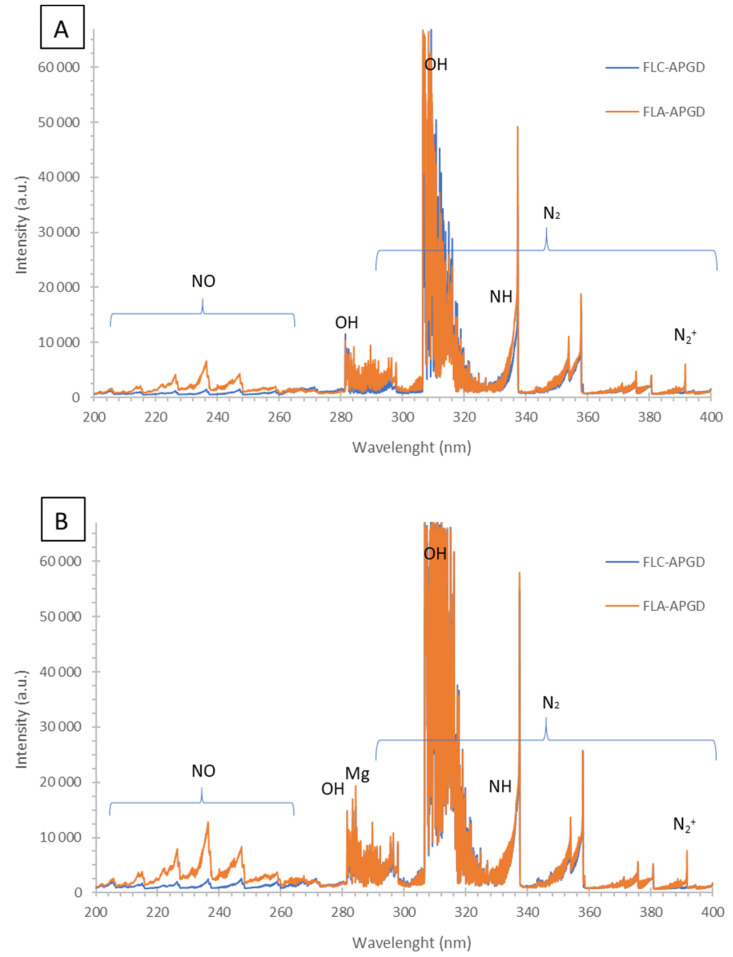
The emission spectra of (**A**) the aqueous *P. ginseng* root extract and (**B**) the aqueous *G. biloba* leave extract during their activation either by FLA-dc-APGD or FLC-dc-APGD.

To identify the RONS responsible for the aqueous plant extracts activation the OES was used. A typical emission spectra of FLA-dc-APGD and FLC-dc-APGD systems, recorded during the activation of both aqueous plant extracts, are presented in Figure 5. As can been seen, the following molecular species NO, OH, N_2_, N_2_^+^ and NH were identified in the spectra. In the 200–260 nm spectral region, several bands of the γ-system (A^2^Σ^+^-X^2^Π) of the NO molecule, that is, (2-0) at 204.7 nm, (1-0) at 214.9 nm, (2-2) at 221.6 nm, (0-0) at 226.3 nm, (0-1) at 236.3 nm, (0-2) at 247.1 nm and (0-3) at 258.7 nm, were identified. This molecule was produced as a result of the chemical reaction between N_2_ coming from the air atmosphere and O radicals, which takes place as follows: N_2_ + O = NO + N. In addition, two strong bands of the OH molecule with the band heads (1-0) at 282.9 and (0-0) at 308.9 nm, belonging to the A^2^Σ-X^2^Π system, were observed for both FLA-dc-APGD and FLC-dc-APGD sources. In opposite to NO, the highly oxidative OH radicals were generated as a result of the water (or its ion) dissociation and/or recombination processes, which occur as follows: H_2_O + e = OH + H + e, H_2_O^+^ + e = OH + H, H_2_O^+^ + H_2_O = H_3_O^+^ + OH. Moreover, in the spectra region from 280 to 400 nm, the strong and numerous bands belonging to the second positive system N_2_ (C^3^Π_u_-B^3^Π_g_), (1-1) and (0-0) bands of the N_2_^+^ molecule (the B^2^Σ^+^_u_-X^2^Σ^+^_g_ system) at 388.4 nm and 391 nm, respectively and the (0-0) band at 336.0 nm of the NH molecule (the A^3^Π-X^3^Σ^-^ system) were excited. Additionally, in the spectra region above 400 nm, the H lines of the Ballmer series (at 434.1, 486.1 and 656.2 nm) as well as the O atomic lines at 777.2, 777.4 and 844.6 nm were noted. These species were from the dissociation processes of water vapour (i.e., H, O) and other reactions, for example, with O_2_, taking place as follows: N + O_2_ = NO + O. Importantly, the Mg I at 285.2 nm as well as K I at 766.5 nm and 769.9 nm lines were also identified as a result of the evaporation and/or sputtering processes of the aqueous *G. biloba* leaves extract. These results were in a good agreement with the EDX analyses (see Section 3.2. for more details).

In order to determine the concentration of the NO molecule in the form of the NO_2_^-^ ions, the colorimetric analyses were performed. It was found that after the impact of FLA-dc-APGD and FLC-dc-APGD on the aqueous plant extracts, the concentration of NO_2_^-^ changed as compared to the untreated aqueous plant extracts (data not shown). In both cases, the concentration of NO_2_^-^ in the FLA-dc-APGD and FLC-dc-APGD treated plant extracts slightly increased as compared to this in the untreated plant extracts. It was noticed that a variety of RONS as well as H radicals were generated in the aqueous plant extracts activated by FLA-dc-APGD or FLC-dc-APGD, as a result of the plasma-liquid interactions [53]. As was suggested by others, the presence of OH, NO and H radicals, hydrogen peroxide (H_2_O_2_), nitrate (NO_3_^−^) and nitrite (NO_2_^−^) ions might be responsible for a partial decomposition of the active organic species occurs in mentioned aqueous plant extracts [41,53]. These species along with long-lived active species could participate in the AuNPs nucleation as well as in their growth in the prepared mixtures. Namely, at low concentrations of the Au (III) ions, H_2_O_2_ molecules likely enhanced their reduction to Au^0^ as a result of the following reaction: 2AuCl_4_^−^ + 3H_2_O_2_ = Au^0^ + 3O_2_ + 6H^+^ + 8Cl^−^, leading to the formation of a relatively high number of the nucleation seeds. At high concentrations of the Au (III) ions, their fast reduction on the surface of AuNPs was likely established, according to the Finke–Watzky two-step mechanism [54]. This would make the synthesized AuNPs larger in size. The produced ions as a result of plasma liquid interaction may also electrostatically stabilize the AuNPs due to the occurrence of the defined charge onto their surface, as was previously suggested by Patel and co-workers [55].

### 3.4. Gold Nanoparticles Stabilization by Active Compounds Originating from Plant Extracts

**Figure 6:**
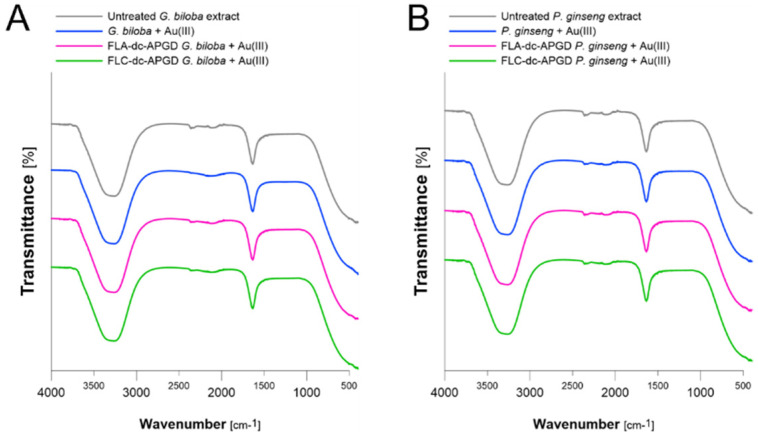
Attenuated total reflectance Fourier transform-infrared spectroscopy (ATR FT-IR) spectra of aqueous (**A**) *G. biloba* and (**B**) *P. ginseng* extracts used for the AuNPs synthesis.

In order to evaluate the interactions between the surface of the synthesized Au nanostructures and active compounds, originating from the applied aqueous plant extracts, ATR FT-IR spectroscopy was used. As can be seen from Figure 6 and Table 3, Amide A and Amide I bands were identified in the ATR FT-IR spectra of the untreated aqueous plant extracts. These bands occurred at 3243, 3254 and 1635 cm^−1^ for the Amide A, Amide I and Amide A bands, respectively. Additionally, these bands were also associated with the presence of N–H and O–H (Amide A) and C=O (Amide I) functional groups. Based on this, it was suspected that these functional groups originated from active compounds present in the prepared aqueous plant extracts, including ginkgolic acids, terpenes trilactiones, flavonoids and proanthocyanidins [32,36]. Interesting, these bands did not disappeared when the aqueous plant extracts were activated by FLC-dc-APGD or FLA-dc-APGD, suggesting that the process did not entirely decomposed the above-mentioned capping agents. However, all the bands in quest slightly shifted as a result of the AuNPs synthesis. This was primarily visible in the case of the Amide A bands, observed for the aqueous *G. biloba* leaves extract activated by both CAPPs. In this case, the above-mentioned bands were shifted to 3245, 3246 and 3245 cm^−1^ after the AuNPs synthesis (Table 3). This suggested that the prepared aqueous plant extracts participated in the formation of AuNPs as well as their further capping and stabilization [17].

### 3.5. Catalytic Activity

The presented green approach was aimed to produce stable-in-time AuNPs that granulometric properties would be controlled by activating the aqueous plant extract with a proper CAPP source, that is, FLA-dc-APGD or FLC-dc-APGD. Because the stability of the resultant Au nanostructures is very important in the processes of the homogenous catalysis, all the samples containing obtained AuNPs were kept at 4 °C for 14 days prior to the tests on their catalytic activity. After this time, it was determined that neither of the samples tends to aggregate. Additionally, because the application of the FLC-dc-APGD-activated plant extracts tended to decrease the average diameter of the AuNPs in the case of the aqueous *P. ginseng* root extract, only the samples containing AuNPs produced with the aid of the FLC-dc-APGD-activated aqueous of *G. biloba* and *P. ginseng* extracts were used for the tests on their catalytic activity.

**Figure 7:**
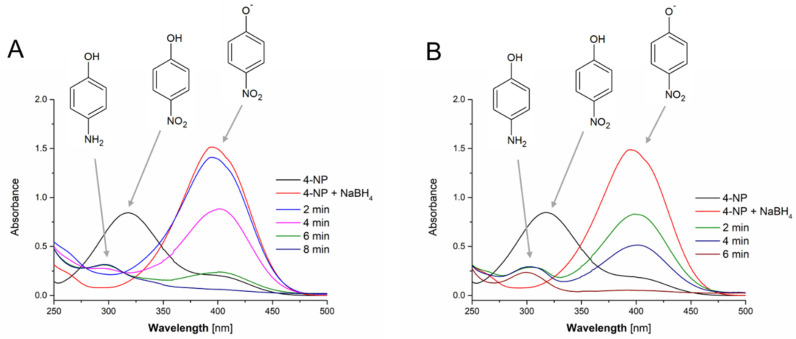
UV/Vis absorption spectra of the catalytic reactions carried out using AuNPs fabricated by (**A**) the aqueous *G. biloba* leaves extract and (**B**) the aqueous *P. ginseng* root extract, both activated by FLC-dc-APGD.

The catalytic reactions were carried out under the supervision of UV/Vis absorption spectrophotometry (Figure 7). The bands at 318 nm was attributed to the presence of 4-NP. After the addition of a NaBH_4_ solution, this band shifted to 400 nm, which was attributed to the reduction of 4-NP and the presence of the 4-nitrophenolate anion. It is well known this reaction requires a catalyst, otherwise, the system remains unchanged [56]. After the addition of AuNPs fabricated using a 500 mg L^−1^ Au(III) solution and both aqueous plant extracts activated by FLC-dc-APGD, the band at 400 nm immediately started to fade (following spectra, Figure 7A,B). Due to the lack of any diffusion limitations (homogenous catalysis), the reaction mixtures responded so rapidly that in case of the application of the aqueous *P. ginseng* root extract with AuNPs it was impossible to record UV/Vis absorption spectrum fast enough before almost half of 4-nitrophenolate anion decomposed. At the same time, another band near 295 nm appeared. This was linked with the formation of 4-aminophenol, indicating the successfulness of the process. Based on the decrease of the absorbance of the band at 400 nm, it was possible to calculate the constant rate (*k*). I was 0.31 and 0.52 s^−1^ for the CAPP-activated aqueous *G. biloba* leave extract and the CAPP-activated aqueous *P. ginseng* root extract, respectively. As a result, the complete reduction of 4-NP was achieved within 8 and 6 min, respectively.

Based on the initial concentration of the Au (III) in the samples (500 mg L^−1^) and assuming their entire reduction to AuNPs, it was possible to recalculate the *k* values into the mass-normalized constant rates *k_m_*_,_ being 2.04 and 3.50 s^−1^mg^−1^ in case of AuNPs obtained using the CAPP-activated aqueous *G. biloba* leaves and *P. ginseng* root extracts, respectively. This enabled to compare the present results for homogenous nanocatalysts with others, being reported in the literature. The suitable summary, setting the proposed approach towards synthesis of homogenous nanocatalysts with comparison of works of other researchers are displayed in Table 4.

Considering the catalytic decomposition of 4-NP using Au-based homogenous catalysts reported in literature, it was concluded that the approach proposed in the present work presented an increased feasibility of the process. FLC-dc-APGD-based activation of the prepared plant extracts allowed not only to efficiently reduce the Au (III) ions to AuNPs and stabilize them but also was responsible for their favourable physiochemical and granulometric characteristics. This enabled the achievement of high *k_m_* values, 10-fold exceeding the *km* values reported for other Au-based nanocatalysts (see Table 4). This was linked with a high reduction efficiency of the Au (III) ions provided by the CAPP-activated plant extracts. The proposed CAPP-based approach, due to its flow-through nature, could be easily adopted for continuous systems, transferring the achieved efficiencies into real-life systems.

## 4. Conclusions

The present work proposes a novel unique approach for AuNPs production suitable for application in homogenous catalysis. The synthetic route involved the CAPP-activation of the aqueous plant extracts of *G. biloba* and *P. ginseng* that were further applied as the reducing and capping agents for the AuNPs fabrication. It was found that the CAPP-treatment might alter the organic species present in the plant extracts that are responsible for the reduction of the Au (III) ions and the stabilization of AuNPs. This, linked with the introduction of the excessive RONS as well as H radicals derived from both FLA-dc-APGD and FLC-dc-APGD, enhanced the reduction of the AuNPs precursor and caused an increased number of the nucleation seeds. As a result, the CAPP-treatment of the aqueous plant extracts resulted in the formation of the colloidal suspensions of AuNPs. These colloidal suspensions were characterized by the increased content of the rod-like and triangular shaped nanostructures, as compared to those produced with the aid of the untreated aqueous plant extracts. The latter ones led to the formation of mainly spherical Au nanostructures. Besides, the FLC-dc-APGD activation of the aqueous *P. ginseng* root extract allowed to obtain the biogenic AuNPs of the smallest average size (14.2 ± 4.4 nm). These AuNPs were selected to test their catalytic activity in the reduction of 4-NP to 4-AP. The so-prepared homogenous nanocatalyst revealed the constant rate of 0.52 s^−1^, which was indeed better as compared to the constant rate of 0.31 s^−1^ that was provided with the AuNPs (29.0 ± 3.5 nm), produced using the untreated aqueous *G. biloba* leave extract. The performance of the presented catalytic systems was particularly tempting as compared to this reported in the literature. The determined mass normalized constant rates were approx. 10-times greater as compared to those given in similar literature reports.

Based on the obtained results, it was concluded that the application of the aqueous *P. ginseng* extract resulted in the formation of smaller in size AuNPs that were much favoured in the catalytic decomposition of 4-NP as compared to the *G. biloba* extract. We believe that the proposed approach might be a very tempting and efficient alternative over other protocols used in the bio-inspired synthesis of the nanocatalysts containing AuNPs.

## Figures and Tables

**Table 1:** Optical properties of AuNPs and absorbance (A) values at the maximum wavelength (λ_max_) of their localized surface plasmon resonance (LSPR) absorption bands.

Type of Plant Extract	Sample	Au(III) Concentration (mg L^−1^)	λ_max_ (nm)	A
*G. biloba*	untreated	125	501.2	0.1444
*G. biloba*	FLA-dc-APGD	125	529.6	0.1897
*G. biloba*	FLC-dc-APGD	125	524.8	0.2010
*G. biloba*	untreated	250	531.6	0.2087
*G. biloba*	FLA-dc-APGD	250	533.6	0.2437
*G. biloba*	FLC-dc-APGD	250	533.6	0.2297
*G. biloba*	untreated	500	536.0	0.3028
*G. biloba*	FLA-dc-APGD	500	535.4	0.4736
*G. biloba*	FLC-dc-APGD	500	544.2	0.4503
*P. ginseng*	untreated	125	546.6	0.0570
*P. ginseng*	FLA-dc-APGD	125	547.8	0.1333
*P. ginseng*	FLC-dc-APGD	125	542.0	0.1465
*P. ginseng*	untreated	250	547.2	0.0519
*P. ginseng*	FLA-dc-APGD	250	555.2	0.0835
*P. ginseng*	FLC-dc-APGD	250	551.4	0.0802
*P. ginseng*	untreated	500	545.2	0.0470
*P. ginseng*	FLA-dc-APGD	500	554.2	0.0766
*P. ginseng*	FLC-dc-APGD	500	546.4	0.0669

**Table 2:** The summary of the shape and size distribution determined for AuNPs synthesized using untreated, FLA-dc-APGD-activated or FLC-dc-APGD-activated aqueous plant extracts. The concentration of the Au (III) ions used in the reaction mixture was 500 mg L^−1^.

	Shape Distribution (%)						Diameter (nm)
Plant Extract	Sample	Spherical	Rod	Triangular	Pentagonal	Hexagonal	
*G. biloba*	untreated	94.6	1.2	3.9	0.7	0.8	15.6 ± 7.0
*G. biloba*	FLA-dc-APGD	87.0	1.8	5.3	2.6	3.3	24.4 ± 4.4
*G. biloba*	FLC-dc-APGD	92.7	2.8	3.9	0.6	0.0	29.0 ± 3.5
*P. ginseng*	untreated	82.6	2.7	10.4	2.3	2.0	16.0 ± 5.5
*P. ginseng*	FLA-dc-APGD	73.2	6.8	14.1	2.0	3.9	21.3 ± 12.3
*P. ginseng*	FLC-dc-APGD	86.8	2.2	8.4	1.3	1.3	14.2 ± 3.8

**Table 3:** ATR FT-IR bands identified for the prepared aqueous plant extracts.

	Functionality	Band Locations (cm^−1^)			
		Untreated Extract	Extract + AuNPs	Extract Treated by FLA-dc-APGD + AuNPs	Extract Treated by FLC-dc-APGD + AuNPs
*G. biloba*	N–H; O–H (Amide A)	3243	3245	3246	3245
	C=O (Amide I)	1635	1634	1635	1635
*P. ginseng*	N–H; O–H (Amide A)	3254	3254	3254	3254
	C=O (Amide I)	1635	1635	1635	1635

**Table 4:** Homogenous nanocatalysts for the catalytic decomposition of 4-nitrophenol.

Nano Catalyst	Synthetic Route	Average Diameter of AuNPs (nm)	*k_m_* (s^−1^ mg^−1^)	Ref.
Au	Bio-based using *S. roxburghian*	17.48	0.43	[10]
	Immobilization on thiol-functionalized halloysite nanotubes	4.20	0.16	[11]
	Diazonium-Au (III) reduced in water	68.20	0.24	[57]
Au-Ag	Borohydride-stabilized	4.60	57.9 (at high Ag concentration)	[58]
Au	*G. biloba* FLC-dc-APGD-activated extract	29.0	2.04	This work
	*P. ginseng* FLC-dc-APGD-activated extract	14.2	3.50	
